# Elucidating the Role of Host Long Non-Coding RNA during Viral Infection: Challenges and Paths Forward

**DOI:** 10.3390/vaccines5040037

**Published:** 2017-10-20

**Authors:** David J. Lemler, Hayden N. Brochu, Fang Yang, Erin A. Harrell, Xinxia Peng

**Affiliations:** 1 Department of Molecular Biomedical Sciences, College of Veterinary Medicine, North Carolina State University, Raleigh, NC 27607, USA; djlemler@ncsu.edu (D.J.L.); hnathan@ncsu.edu (H.N.B.); fyang6@ncsu.edu (F.Y.); eaegan@ncsu.edu (E.A.H.); 2 Bioinformatics Graduate Program, North Carolina State University, Raleigh, NC 27695, USA; 3 Bioinformatics Research Center, North Carolina State University, Raleigh, NC 27695, USA

**Keywords:** lncRNA, next-generation sequencing, high-throughput analysis, transcriptomics, RNA-seq

## Abstract

Research over the past decade has clearly shown that long non-coding RNAs (lncRNAs) are functional. Many lncRNAs can be related to immunity and the host response to viral infection, but their specific functions remain largely elusive. The vast majority of lncRNAs are annotated with extremely limited knowledge and tend to be expressed at low levels, making ad hoc experimentation difficult. Changes to lncRNA expression during infection can be systematically profiled using deep sequencing; however, this often produces an intractable number of candidate lncRNAs, leaving no clear path forward. For these reasons, it is especially important to prioritize lncRNAs into high-confidence “hits” by utilizing multiple methodologies. Large scale perturbation studies may be used to screen lncRNAs involved in phenotypes of interest, such as resistance to viral infection. Single cell transcriptome sequencing quantifies cell-type specific lncRNAs that are less abundant in a mixture. When coupled with iterative experimental validations, new computational strategies for efficiently integrating orthogonal high-throughput data will likely be the driver for elucidating the functional role of lncRNAs during viral infection. This review highlights new high-throughput technologies and discusses the potential for integrative computational analysis to streamline the identification of infection-related lncRNAs and unveil novel targets for antiviral therapeutics.

## 1. Introduction

Viral infections remain a major world-wide concern. Even though we have all but eradicated some once deadly viruses, many still elude effective treatment. HIV-1 is becoming a chronic disease since current anti-retroviral therapy can suppress the infection but cannot clear the virus. Influenza is still a major global concern that evolves rapidly and kills thousands of individuals every year. As shown by recent examples, like Zika and Middle East Respiratory Syndrome Coronavirus (MERS-CoV), the threat of emerging viral infections is constant. A better understanding of virus and host interactions is needed to accurately define viral pathogenesis and to rapidly develop new therapies.

Long non-coding RNAs (lncRNAs), a new class of transcripts, have recently garnered interest in the field of infection, as studies of the host response to viral infection typically focus on protein-coding genes. LncRNAs are defined as RNAs greater than 200 nucleotides with insignificant coding potential, i.e., noncoding, but they can have very diverse regulatory functions ranging from active transcriptional regulation to epigenetics. Compared to the small number of proteins that are druggable, a large number of lncRNAs offer many new potential targets as they may be easily and accurately targeted using sequence specific oligonucleotides, and they are more cell type- and tissue- specific than coding genes in general. We and others have documented the association of lncRNAs in viral infections and innate immunity [1,2,3,4,5]. The functions of numerous lncRNAs in host immune responses have also been extensively reviewed [6,7,8]. However, most lncRNAs, including those associated with viral infections, still lack detailed functional characterization.

First, we summarize our current understanding of the diverse functions of lncRNAs and their potential relevance to viral infection (Section 2). The analysis of lncRNA functions in viral infections comes with unique challenges, including their overwhelmingly low expression abundance, lack of sequence conservation, and limited annotation. To better address these challenges, we broadly outline the key steps to be taken to investigate infection-associated lncRNAs: discovery (Section 3.1), prioritization (Section 3.2, Section 3.3 and Section 3.4), and validation (Section 3.5). We discuss strategies, including existing and emerging technologies, for addressing challenges that researchers may encounter during each step. We conclude with suggestions for new developments needed to rapidly identify infection-related lncRNAs and potentially novel anti-viral targets. While our primary interest is viral infection, the general ideas we present here can also facilitate the studies of lncRNAs in other areas.

## 2. Functional Diversity of lncRNAs and Their Involvement in Viral Infections

LncRNAs are generated using the same machinery and components as mRNA (and other ncRNA), may possess 5′ caps, and may also be polyadenylated at their 3′ ends. Their main distinction from mRNA is the lack of a significant open reading frame. Unlike coding genes, lncRNAs tend to be poorly conserved at the primary sequence level [9,10]. LncRNAs are also known to have lower and more tissue- or cell-type specific expression than coding genes [11]. LncRNAs permeate the human genome and interdigitate coding regions, but are often dismissed as transcriptional noise due to low expression levels and the lack of primary sequence conservation. Expressed lncRNA genes have typical histone modifications, exhibit canonical splice site signals, and can produce alternative transcripts [11,12]. They localize both in the cytoplasm and the nucleus, suggesting roles in the epigenetic modification of chromatin and regulation of gene expression. Their structural architecture has been described as modular and multi-domained, allowing lncRNAs to form conformational switches and simultaneously interact with mRNAs, DNA, and proteins [13]. The functional roles of these RNAs are diverse and, due to their typically low sequence conservation, are believed to rely on secondary and tertiary structures.

Much of the initial interest in lncRNAs stems from the discovery of Xist and its involvement in X chromosome inactivation (XCI), a process that has been detailed in a number of extensive reviews over the years [14,15,16,17]. Though the majority of lncRNAs were considered “junk RNA” and transcriptional noise, lncRNAs have now been shown to regulate methylation [18,19], chromatin remodeling [20], and alternative splicing [21], as well as imprinting and dosage compensation [17]. Despite the lack of functional characterization of most lncRNAs, many have been functionally implicated in viral infections [3]. Of the many functions described above, several are known to be exhibited by lncRNAs associated with viral infections, e.g., paraspeckle formation [4] and pre-mRNA splicing [22]. Such lncRNAs are often involved in the host immune response, but many are also virally-encoded. In Table 1, we list several better characterized lncRNAs that are associated with commonly studied viruses, including influenza and herpes. Recent reviews by Ding et al. [23], Mumtaz et al. [6], Valadkhan et al. [7], and Liu et al. [8] highlight many of the extensively annotated host- and virally-encoded lncRNAs.

### 2.1. Epigenetic Regulation and Promotion of Viral Latency

LncRNAs have been established as significant players in epigenetic regulation. Several studies have characterized associations between lncRNAs and DNA methyltransferases (DNMTs): DNMT1 [31,32], DNMT3a [33], and DNMT3b [34]. The interactions between lncRNAs, various histone modifiers, and chromatin remodeling complexes have been extensively reviewed, among which, the HOXA and HOXC clusters are particularly well-studied [35,36]. The biological functionality of lncRNAs from the HOXA and other loci as recruiting factors of major complexes is well-described [37,38,39]. A comprehensive review by Betancur et al. [20] underscores the pervasive interactions between lncRNAs and complexes such as PRC1, PRC2, MLL1, and BAF. The regulation of expression via chromatin looping and nucleosome positioning is also an established function of lncRNAs [12]. LncRNA methylation functionality and their involvement in developmental processes and disease are highlighted in several reviews [18,19,40].

Several host- and virally-encoded lncRNAs have also been shown to function epigenetically. For example, PAN RNA (Table 1) has been implicated in the transcriptional inhibition of the IFN cascade and is known to alter gene expression by adding or removing H3K27me3 marks through interaction with the PRC2 complex and histone methyltransferase MLL2 [8]. Virally-encoded lncRNAs have also been shown to interact with DNMTs and chromatin remodeling complexes. Rossetto et al. demonstrated that a human cytomegalovirus (HCMV) lncRNA interacts with Polycomb Repressive Complex 2 (PRC2) during latency to inhibit host transcription [41]. Saayman et al. [29] identified an HIV-1-encoded antisense lncRNA (Table 1) that regulates viral transcription via direct interaction with a chromatin remodeling complex consisting of DNMT3a, EZH2, and HDAC-1. The HIV-1-encoded antisense lncRNA also interacts with PRC2, inhibiting viral transcription and affecting nucleosome assembly [42]. Both of these viral lncRNAs have been implicated in the promotion and maintenance of early latency of their respective viral infections [41,42]. Moving forward, the extent of the relationship between epigenetic regulation and viral latency resulting from lncRNA expression needs to be further investigated.

The role of lncRNAs in epigenetic regulation has also been described through the concept of extra-coding RNA (ecRNA). The term was first introduced by Di Ruscio et al. [32] upon the identification of a ncRNA (ecCEBPA) in humans that contained the entire CEBPA pre-mRNA sequence along with “extra” up- and down-stream regions. ecCEBPA blocks the methylation of the CEBPA promoter region via a direct interaction with DNA methyltransferases (DNMTs). The expression of ecCEBPA was later associated with neuronal gene promoter methylation [33]. While the validity of this class of RNA is still open for debate due to similarities with pre-mRNA, their potential involvement with the host immune response might be of great interest.

### 2.2. Scaffolding and Nuclear Localization

Biological scaffolding is a function typically reserved for proteins; however, it has recently been identified as a function of lncRNAs. In plants, RNA polymerase V-dependent lncRNAs are known to recruit AGO4 in the canonical RNA-directed DNA methylation (RdDM) pathway [43]. The functional role of lncRNAs has been expanded to include stepwise binding to IDN2 and DRM2 in addition to AGO4, characterizing lncRNAs as a lynchpin of the RdDM pathway [12]. The function of lncRNAs as scaffolds is also described in the formation of oddly-formed nuclear compartments in mammalian cells, known as paraspeckles. Two examples are the HIV-associated lncRNAs NEAT1 (Table 1) and MALAT1. While NEAT1 has been established as an essential structural scaffold in the maintenance of nuclear paraspeckles, MALAT1 is not required [44]. The role of these lncRNAs in paraspeckle formation has been extensively reviewed [44,45,46,47]. The knockdown of NEAT1 in HIV-1-infected T cells demonstrated that the maintenance of nuclear paraspeckles by NEAT1 is associated with HIV-1 replication [2]. More recently, NEAT1 was shown to promote IFN production in response to Hantaan virus (HTNV) infection by localizing SFPQ (splicing factor proline- and glutamine-rich protein) to nuclear paraspeckles [24]. In vitro and in vivo inhibition of NEAT1 expression suppressed the host immune response. Conversely, ectopic expression increased IFN-β production and inhibited HTNV replication. These results indicate that NEAT1 can modulate the host immune response by the localization of RNA-binding proteins to paraspeckles. Whether or not NEAT1 exhibits this functionality in response to other viral infections needs to be investigated.

### 2.3. Transcriptional Regulation of mRNA via miRNA Sponges

LncRNAs can also act as sponges for miRNA and prevent the miRNA-mediated degradation of target transcripts. The mode of this function has been described through the competing endogenous RNA (ceRNA) hypothesis and has been extensively reviewed [48,49]. LncRNAs have been functionally described as ceRNAs in the progression of gastric cancer [50] and hepatocellular carcinoma [51]. The involvement of ceRNAs, such as lincROR, HOTAIR, and BARD1 9′L, in the initiation and progression of various cancers has also been well-documented and reviewed [52,53]. There is also evidence that virally-encoded lncRNAs function as miRNA sponges. When studying Herpesvirus saimiri (HVS), Cazalla et al. [54] identified sequence complementarity between HVS-encoded lncRNAs (HSUR1 and HSUR2) and host miRNAs expressed in T cells, and confirmed the downregulation of miRNA-27 as a result. Guo et al. [55] recently analyzed the functional roles of miRNAs in HVS-transformed T cells, revealing that miRNA-27 directly downregulates several proteins in the T cell receptor signaling pathway. A review by Tavanez et al. [56] further describes the function of HSUR1 and HSUR2 and highlights the susceptibility of host miRNAs in viral infections. This interplay of virally-encoded lncRNAs and host miRNAs may prove to be an essential function of viral pathogenesis.

### 2.4. Alternative Splicing

The notion that lncRNA can affect alternative splicing stems from the fact that these RNAs are often found in complex loci that also contain protein-coding genes. A recent study has shown that the highly conserved human h5S-OT lncRNA expressed within the 5S rDNA locus can impact alternative splicing by interacting with U2AF65, a core splicing factor that binds to intron-exon junctions [57]. An anti-Alu element in the 3′ end mimics the polypyrimidine tract necessary for the recruitment of splicing factors, resulting in the targeting of introns containing Alu elements and downstream exon inclusion [57]. Previously associated with HIV-1 infection [3], MALAT1 has been shown to regulate trans-acting pre-mRNA splicing factors in cancer cells, specifically those in the SR protein family [22]. Extensive reviews on MALAT1 [58], RNA-guided mechanisms in alternative splicing [21], and several other disease-related lncRNAs experimentally linked to alternative splicing [59,60] are available for further reading.

## 3. Discovery, Prioritization, and Validation of lncRNAs

In the previous section, we discussed some of the characteristics and functions of lncRNAs. Due to their unique nature, methodologies developed for the interrogation of coding genes may not be applicable or may require modification to adequately investigate lncRNAs. This section will discuss selected methods for the discovery, prioritization, and validation of lncRNAs. We begin with a discussion of the modifications to RNA-seq that allow for the detection of lncRNAs (Section 3.1), which represents the discovery phase for determining the association of lncRNAs with viral infections. The prioritization phase focuses on computational methods for ranking lncRNAs based on differential expression and genomic context (Section 3.2). Section 3.3 delves further into the genomic context of lncRNAs by incorporating evolutionary analyses to aid in the identification of function. An alternative or complementary strategy for identifying lncRNA function employs large scale in vitro screening studies (Section 3.4). These screens are capable of directly associating genes with the phenotype of interest while providing support for computationally derived results. Finally, Section 3.5 highlights important considerations for validation that arise as a result of the unique features of lncRNAs. Together, these methods represent a comprehensive strategy for identifying the involvement of novel lncRNAs in viral infection while simultaneously utilizing all available data to ascertain function.

### 3.1. Discovering Viral Infection-Related lncRNAs: The Different Flavors of Transcriptome Deep Sequencing

During viral infection there are a multitude of changes throughout the transcriptome, which includes lncRNAs. Therefore, a natural choice for discovering specific lncRNAs important for infections is to systematically profile lncRNA changes in response to infection. While multiple methods for the detection of lncRNAs are currently available, as highlighted in [61], transcriptome deep sequencing (RNA-seq) is likely the most preferred and widely used, in part due to its broad applicability and accessibility. More importantly, RNA-seq directly sequences actual transcripts, regardless of whether or not the reference genomic sequences exist [62]. This is especially relevant as many viral infections occur in non-model organisms that do not have fully sequenced genomes or well-annotated with lncRNAs. Though RNA-seq is available as a standard service from core facilities, there are still specific considerations in terms of studying lncRNAs.

#### 3.1.1. Total RNA vs. mRNA

Standard RNA-seq focuses on mRNAs (mRNA-seq) by enriching transcripts with poly(A) tails using approaches like poly(dT) coated magnetic beads. This would be less desirable for lncRNA analysis as many lncRNAs lack poly(A) tails. Alternatively, the expression of both poly(A+) (mostly mature transcripts of coding genes) and poly(A-) (mostly non-coding RNAs) can be captured by the sequencing of total RNAs (Total RNA-seq), as shown by our studies [1,3] and others. Limitations to Total RNA-seq, on the other hand, include the requirement of millions of additional sequencing reads to achieve a comparable coverage as mRNA-seq, and the difficulties in quantifying individual isoforms due to the coverage of immature pre-mRNAs. In spite of this, we have used this technique to identify non-coding RNAs differentially expressed during HIV-1 [3], SARS coronavirus [1], and influenza infections [63]. Further, total RNAs may be separated into mRNA enriched and mRNA depleted fractions [32], allowing for coding and noncoding transcripts to be analyzed independently. In any case, special attention should be paid to the removal of extremely abundantly transcripts like ribosomal RNAs in total RNAs and hemoglobin transcripts in whole blood samples.

LncRNAs are frequently expressed at very low levels. Total RNA-seq or the sequencing of mRNA depleted RNAs may still be insufficient for the detection of lowly expressed lncRNAs [61,64]. Instead, RNA capture sequencing (CaptureSeq, CAP-seq) uses custom-designed, hybridization-based oligonucleotide probes to capture and enrich genes or regions of interest [64]. The probes are designed to target the transcripts of interest, while untargeted species are washed away [64]. The enriched fraction is subjected to sequencing, resulting in greatly increased target coverage [64]. When sufficient reference sequence information is available for probe design, this technique may ease detailed transcript assembly and abundance quantification [64]. In particular, this method might be effective for profiling a list of target lncRNAs across a large number of samples.

#### 3.1.2. Computational Considerations for Identifying lncRNAs from RNA-seq Data

Like any other study, the described RNA-seq analysis requires necessary computational infrastructure and bioinformatics expertise for data storage, processing, and statistical analysis. Ideally, this is planned out before the start of the study, and may be accomplished in-house, in-collaboration, or through core services. Particularly, the annotation of lncRNA genes and its completeness should be examined carefully. For viral infections in well-studied systems like humans or mice, there are large numbers of annotated lncRNAs. For example, there are 14,720 human lncRNA genes and 8980 mouse lncRNA genes in the current Ensembl annotation (release 90.38). In these cases, lncRNA expression analysis can be carried out simultaneously just as all other coding genes. However, after quality control, it would be very beneficial to align cleaned sequencing reads to viral reference sequences to verify the identity of infected samples and the percentage of total reads mapped to viruses.

For other organisms with draft genome assemblies, it is likely that the annotation of lncRNA genes is largely incomplete. Therefore, it would be important to annotate lncRNA transcripts ab initio, using the collected RNA-seq data in combination with other available sequencing data. For example, we were able to expand the ferret genome annotation with about 40,000 intergenic loci, which were enriched with polyadenylated and non-polyadenylated intergenic ncRNAs [3]. Similarly, we recovered thousands of ncRNA enriched intergenic loci in both rhesus macaque and cynomolgus macaque genomes [3]. Typically, this lncRNA annotation can be accomplished by first aligning sequencing reads to the species matched reference genome assembly using gapped aligners such as TopHat [65] or STAR [66]. The aligned reads can then be assembled into transcripts using tools such as Cufflinks [67], Scripture [68], or StringTie [69]. Depending on the completeness of the reference genome assembly, unmapped short reads may also be assembled de novo into full-length transcripts or longer transcript fragments using tools like Trinity [70]. These assembled transcript sequences can now be aligned back to the original genome assembly or simply added to the existing reference sequences and updated annotations.

Transcripts assembled from RNA-seq data consist of known as well as novel transcripts. Tools like Cuffcompare, part of the Cufflinks package [67], may be used to compare transcripts against reference annotations to separate novel from known genes. In order to focus on lncRNAs, it is important to filter out unwanted transcripts such as single-exon transcripts and transcripts of less than 200 nucleotides [71]. Next, transcripts with protein-coding potential can be assessed using various conservation-based computational tools, such as the phylogenetic codon substitution frequency (PhyloCSF) [72]. This can be done alone or in combination with non-conservation-based computational tools, such as a coding potential calculator (CPC) [73], coding potential assessment tool (CPAT) [74], and LncRNApred [71,75,76]. To further filter out potential protein coding transcripts, tools like HMMER software can be used to sort out transcripts that encode protein domains [71,77]. The remaining transcripts are putative lncRNAs and can be incorporated into further RNA-seq data analysis. It is also possible to perform lncRNA analysis for species without a reference genome assembly by complete de novo transcript assembly using various tools like Trinity [70] or Trans-ABySS [78] followed by similar filtering steps as described. Since there is no reference genome, lncRNA quantification should use transcript alignment-based methods like RSEM [79] or alignment-free methods like Salmon [80].

While it is not covered here, we want to emphasize that as with any other experiments, proper experimental design should be carefully conducted before the start of infections and RNA-seq analysis.

#### 3.1.3. Singling Out Cells in Lieu of Bulk Analysis

An exciting new development of RNA-seq is the analysis of large numbers of individual cell transcriptomes [81]. Transcriptome studies of viral infection are often conducted on samples of mixed cell populations, resulting in an average population-level expression for each transcript [82]. However, in doing so, the distinct functions of different cells may be overlooked. Lung tissue from an influenza virus infected individual can be a mixture of infiltrated immune cells, infected epithelial cells, uninfected epithelial cells, and many other types of cells. Similarly, a solid tumor is composed of various types of infiltrating immune cells in addition to a heterogeneous mixture of cancerous cells carrying a range of mutations and gene expression patterns [83]. To address this cell-to-cell heterogeneity on a genomic scale, researchers turn to single-cell RNA-seq (scRNA-seq). Aside from the isolation of single cells and amplification of small volumes of RNA, scRNA-seq is very similar to conventional bulk RNA-seq [82]. Although scRNA-seq has many advantages when compared with bulk RNA-seq, researchers should carefully consider multiple factors when they plan to use this technology for lncRNA analysis. First, existing scRNA-seq protocols can only capture polyadenylated transcripts, so many of the ploy(A-) lncRNAs will be missed. Second, due to the limited materials, the detection of lowly expressed transcripts like lncRNAs might be less sensitive in single cells than bulk samples [84]. Third, multiple technical factors like the biosafety measures for handling infectious materials and the breakdown of tissues with enzymes during cell dissociation used in most scRNA-seq protocols may impact transcriptional profiles [84]. Notwithstanding these caveats, scRNA-seq has the potential to enable the identification of highly cell type specific lncRNAs that escape identification through current bulk sequencing strategies. The applications of scRNA-seq in large scale perturbation studies will be discussed further in Section 3.4.

### 3.2. Prioritization of Infection-Related lncRNAs by Computational Prediction

A typical RNA-seq analysis of virus infected samples may identify hundreds if not thousands of lncRNAs that respond to infection. One of the more challenging analytical steps is the prioritization of these identified lncRNAs, i.e., the identification of a small subset of lncRNAs that may play more important roles in the phenotype of interest. We see from Table 1 that many of the annotated lncRNAs have been researched through ad hoc experimentation, whereas potentially novel lncRNAs are overlooked in favor of those that have been previously studied. Due to the lack of lncRNA functional annotations, researchers may also simply limit downstream analysis to a few lncRNAs that have significant differential expression and/or are highly abundant. Given the limitations of this strategy, we recommend that additional computational analyses may help to guide this prioritization.

#### 3.2.1. Prediction of lncRNA Function by “Guilt-by-Association”

The prediction of individual lncRNA functions is often based on “guilt-by-association” analysis, wherein tightly co-expressed lncRNA and coding transcripts are assumed to share similar functions [85,86,87,88,89]. Therefore, these lncRNAs may be inferred with the same functions as those annotated coding genes. This prediction starts with expression data that simultaneously profiles both coding genes and lncRNAs across multiple conditions. This expression data can be the same RNA-seq data used for identifying infection-related lncRNAs, or a complementary RNA-seq data relevant to the infection, or both. As an example, an independent human tissue compendium of RNA-seq data was used to infer the functions for a set of HIV infection-related human lncRNAs [3]. In another example, the functions of infection-related lncRNAs were directly inferred from the same RNA-seq data from mouse lung samples infected with either influenza A virus or severe acute respiratory syndrome coronavirus (SARS-CoV) [63]. Next, the choice of a suitable method for calculating co-expressions may be guided by an initial assessment of co-expressions of genes annotated with similar functions, as illustrated in [63].

There are multiple approaches for inferring putative lncRNA functions from co-expressed coding genes. As shown in [3,63], for each lncRNA of interest, all detected coding genes can be ranked by their correlation coefficients with the lncRNA. The enrichment of annotated gene sets, such as pathways and biological processes, in this ranked list of coding genes can be analyzed using commonly used methods, including gene set enrichment analysis (GSEA) [90], to obtain the functions that are highly correlated with the specific lncRNA.

Alternatively, co-expressed lncRNAs and coding genes can be determined by clustering methods. Genome-wide clustering of similar gene expression profiles extrapolates lncRNA function based on known gene functions in the same cluster, where transcripts in the same cluster are considered to be co-regulated [91]. The commonly used clustering methods include hierarchical clustering, k-means clustering, and self-organizing maps (SOMs) [87,91,92,93]. Similarly, a network-based approach can be utilized to predict the functions of lncRNAs from the known functions within the same network [91]. For example, a popular method called weighted gene co-expression analysis (WGCNA) [94] can be used to build gene co-expression networks using the RNA-seq data from virus infected samples. It first constructs a gene-gene network based on similar gene expression profiles, then divides genes with a similar expression into groups of genes or network modules. These modules of both lncRNAs and coding genes can then be annotated with enriched biological functions and associated with clinical traits. LncRNAs occupying key points (hubs or bottlenecks) in the derived networks may likely play important regulatory roles. As shown in [63], some of the identified lncRNAs may regulate the interferon response to viral infection.

#### 3.2.2. Prediction of lncRNA Function Based on Local Genomic Context

Another strategy for inferring putative functions of lncRNAs is to examine protein-coding genes located near lncRNAs of interest, as lncRNAs may have cis-regulatory effects on flanking genes [86,95]. For example, in an early study we found that during SARS-CoV infection the changes in the expression of neighbor coding genes in mouse lungs were significantly associated with those of the corresponding lncRNAs, suggesting those lncRNAs may modulate host responses through neighboring coding genes [1]. Similarly, in a follow-up study with a larger number of samples, we also observed positive correlations between potential cis-regulatory lncRNAs and coding gene neighbors, indicating that some lncRNAs have transcriptional “enhancer-like” functions during viral infections [63]. Therefore, it is useful to prioritize lncRNAs proximal to coding genes, as they may have a higher potential for regulatory functions.

When portions of lncRNAs overlap a coding gene, it is expected that this region be conserved and under a strong selective pressure. A recent study found that the evolutionary age, overlapping configuration, and local genomic environment of an lncRNA-coding gene pair influence the expression correlation of the pair [96]. While there needs to be further research of lncRNA-coding pairs in more well-annotated genomes, it is clear that positional information is important in understanding their functional relationship. As we will discuss in Section 3.5, it is crucial to consider lncRNA baseline expression relative to its coding counterpart when performing the knockdown or overexpression of such an lncRNA.

### 3.3. Identifying Functional lncRNAs Using Evolutionary Analysis

When assessing the function of a gene, including lncRNAs, it is imperative that it is done in the context of evolution. Homology searches leverage annotation from closely related organisms in order to infer the function of novel lncRNAs. The existence of homology often indicates that there is some selective pressure for the lncRNA in question, thereby implying functional significance. However, lncRNAs pose computational challenges when it comes to identifying and ascertaining cross-species homology. LncRNAs have been previously categorized into subclasses, each with functions that reflect their evolutionary conservation [9]. For example, lncRNAs that occlude the transcription of coding genes through their own transcription typically lack sequence and structural conservation outside of those regions overlapping the promoter region of the coding gene. The heterogeneity of lncRNAs is clear when considering not just the variety of their functions, but also their wide range of conservation. Therefore, all forms of conservation: sequence, syntenic, and structural, must be considered to effectively conduct homology searches of lncRNAs.

#### 3.3.1. Incorporating Synteny in Sequence Homology Searches

It is well-understood that lncRNAs often possess very little conservation at the primary sequence level [9,10]. For example, it has been estimated that there is only 22% sequence identity between orthologous human and mouse lncRNAs [97]. Furthermore, there are only a few examples of known lncRNAs with sequence conservation tantamount to coding genes, e.g., TUNA and MALAT1 [10]. The disparity between lncRNA exon and mRNA exon sequence conservation suggests that there is little selective constraint at this level [9]. There is, however, negative selection in promoter regions, which is one of the few commonalities across the lncRNA landscape [10,98]. Despite the lack of sequence conservation across species, there still exists many cases of functional conservation, suggesting the conservation of higher order structures.

Positional information can aid sequence homology searches, whereby the homology of lncRNAs is inferred by their relative positioning near orthologous genes. This syntenic conservation can help overcome the shortcomings of lncRNA sequence homology searches [9]. A recent study identified lncRNAs from Arabidopsis thaliana that were syntenic with high sequence divergence in two closely related plant species [99], which validates the assertion that synteny can be more informative than the sequences themselves. Moreover, the existence of syntenic conservation confers functional significance, as it indicates cross-species selective pressure. This additional conserved feature has been employed in two recently developed tools, slncky [100] and Evolinc [101]. When tested on a set of known orthologous human-mouse lncRNAs, slncky successfully identified the vast majority as homologous and increased the size of this orthologous set by roughly 8%. Furthermore, these computational analyses may help investigators decide which lncRNA targets to study further, since conserved lncRNAs can potentially be further studied in other species, especially using in vivo models.

#### 3.3.2. Using Structural Conservation to Functionally Annotate lncRNAs

The lack of sequence conservation among most lncRNAs has also prompted researchers to look at higher-order structures to infer functional significance. It is commonly understood that canonical RNAs, such as rRNA and tRNA, can form complex secondary and tertiary structures. There is now mounting evidence that lncRNAs, unlike shorter ncRNAs, assume higher-order structures with functional significance. For example, MALAT1 is known to have a highly conserved 3′ cloverleaf structure, one of several structures described by Nitsche et al. [10]. The most common methods for predicting the secondary and tertiary structure of lncRNA are extensively reviewed by Yan et al. [102]. Pfold [103] and Foldalign [104] are two examples of methods that use multiple sequence alignments to predict the RNA secondary structure. Mfold [105] and RNAfold [106] are popular examples of minimum free energy models. Alternatively, CMfinder [107] is an expectation maximum algorithm that uses a heuristic approach to search for RNA motifs, which is not constrained to sequence-based alignments.

Many of these approaches fall short of identifying conserved lncRNA structures, as their focus lies primarily at the sequence level. To overcome the low sequence identity observed among many ncRNAs, a recent approach has made use of CMfinder in a computational pipeline that makes structural alignments to successfully capture conserved RNA structures (CRSs) [108]. The study also showed the functional significance of these CRSs, showing an enrichment in gene regulatory regions as well as overlap with RBP binding sites. The method predicted CRSs for a staggering 22% of lncRNAs annotated in GENCODE v25, showing a higher density of CRSs at the 5′ end of lncRNAs. Interestingly, the majority of lncRNAs still lack CRSs. This could be a biological phenomenon of lncRNAs; however, it could also be due to the implementation of the tool. The search strategy encompasses many divergent species, making the search for conserved CRSs highly stringent. If the search space were limited to more closely related species, there could be a higher rate of CRS identification. Future studies may devise a tiered strategy consisting of an initial broad search followed by more narrow searches of less divergent species.

### 3.4. Large Scale Perturbation Studies for Probing lncRNA Functions

Considering the large number of lncRNAs that are lacking functional annotation, large scale perturbation will be another attractive strategy for probing their roles in viral infection. As shown by earlier studies [109,110,111], traditional large scale screening of host factors related to viral infection has been based on RNA interference (RNAi). Though RNAi-based perturbation can be applied to investigations of a large number of lncRNAs [112], targeting lncRNA in this manner may not be optimal. Many lncRNA exert their effect in the nucleus, NEAT1 and MALAT1 for example [113], whereas shRNAs are processed through DICER, which is mainly cytoplasmic. While nuclear DICER activity has been demonstrated, its activity may be cell type specific [113]. Additionally, the act of transcription is often sufficient to observe the downstream effects of lncRNAs [114]. In this case, targeting the transcript may not produce the desired functional effect. Finally, the off-target effects of RNAi are well-documented [115].

As summarized in [116], the CRISPR-Cas9 system has recently emerged as the leading technique for screening host factors important for different viral replications. These screens targeted coding genes based on the loss-of-functions induced by small indels created by the CRISPR-Cas9 system. New modifications are rapidly advancing this approach for screening non-coding RNAs. For example, considering that indels caused by a single cut from Cas9 in non-coding regions are unlikely to produce a functional knockout, Zhu et al. [117] reported a high-throughput method to produce large deletions of non-coding DNA that is based on a lentiviral paired guide RNA (pgRNA) library. Using this screening method, they identified 51 human lncRNAs that can positively or negatively regulate human cancer cell growth and validated nine of the 51 lncRNA hits using multiple orthogonal techniques.

Alternatively, Cas9 is mutated to an endonuclease-deficient form (dCas9) and fused to a repressive Krüppel associated box (KRAB) motif preventing cytotoxicity due to DNA double-strand breaks [118] and inhibiting target gene expression [119]. The guide RNA (gRNA) library is produced via oligonucleotide printing and packaged into a lentiviral pool. Cell lines expressing dCas9-KRAB are transduced with the gRNA library in order to achieve a single gRNA copy per cell [120]. The resulting dCas9-KRAB inhibits transcription by directly blocking transcription machinery or through the activity of an effector domain, in this case KRAB [119]. At the conclusion of the treatment of interest, barcoded gRNAs are PCR amplified and deep sequenced to identify gRNA enrichment and thereby genes associated with susceptibility to the treatment [120].

Gilbert et al. [121] established a proof-of-concept by combining dCas9-KRAB with a gRNA library in K562 cells. The inhibition of coding genes in this manner resulted in high specificity and low off-target effects [121]. Additionally, a small library (six genes targeted by three gRNAs each) was used to target lncRNAs, resulting in an at least 80% knockdown of target genes as determined by qPCR [121]. The Weissman group expanded on that small library with a large scale CRISPRi library screen of lncRNA in seven different cell lines consisting of a gRNA library targeting over 16,000 lncRNA genes [122]. Between 28 and 438 lncRNAs were determined to cause a growth phenotype [122]. Among these lncRNAs was LINC00263, which was associated with a negative growth phenotype. LINC00263 was used to demonstrate the cell type specificity of lncRNA by showing that there is very low correlation between transcript abundance and the presence of a phenotype. Additionally, the treatment of all seven cell lines with LINC00263 antisense oligonucleotides retards proliferation differently in each cell line, further demonstrating the cell type specificity of LINC00263 and lncRNAs in general.

#### 3.4.1. Gain-Of-Function Library Screen

Often, lncRNA genes are not detectably expressed, or they may be repressed by a disease state. This limited expression of lncRNAs makes detecting meaningful changes by inhibitory assays difficult, if not impossible. Several CRISPR-Cas9 methods have been developed to induce gene expression [118,123,124,125], which might be able to address this issue. These CRISPR activation proteins rely on the same endonuclease-deficient Cas9 described above. dCas9-SunTag is fused to a peptide epitope which recruits co-expressed VP64 activation domains [123]. A high-throughput gRNA library screen using this system in K562 cells was able to identify gene activation affecting cell growth in K562 cells as proof-of-principle [121]. Synergistic Activation Mediator (SAM) and dCas9-VPR are fused to multiple activation domains [124,125]. SAM was used to screen code genes for gain-of-function resulting in BRAF inhibitor resistance in A375 melanoma cells [125]. This screen identified 13 genes that were then individually confirmed at the transcript and protein level. This system was also used successfully to identify the lncRNA AK023948 as a positive regulator of AKT [126]. dCas9-VPR was shown to enhance the activation of the non-coding gene MIAT, in addition to other genes [124]. These developments highlight the potential of using dCas9-based activators to study lncRNA functions during viral infection.

#### 3.4.2. New Developments: Multiplexed Library Screen and Single cell Library Screen

An exciting development for these screening methods is to inhibit or activate two or more target genes within the same single cells [127,128], as it allows the interrogation of complex gene-gene interactions. Currently, the assay relies on the expression of multiple gRNAs from a single plasmid or the expression of multiple plasmids, each with a single gRNA [127,128]. It is also possible to combine gRNAs such that genes are activated and inhibited in parallel [129]. Combining large libraries is a complex task. Not only does this generate a tremendous amount of complex data, but the cost increases considerably with the library size. One method employed to mitigate these issues is to conduct preliminary screening with a single unbiased library. The resulting hits from the initial screen can then be used to produce a smaller library for the second round screening. The second round of screening could involve comparing the smaller library against itself or against the larger unbiased library [120,127].

Another key consideration for designing large scale screens is the choice of readout for approximating the phenotypes of interest. Using single cell sequencing (scRNA-seq) as a readout represents a potential paradigm shift [128,130,131]. Typically, the readout is relatively simple such as cell proliferation or survival, or some sortable marker proteins, which can be very limiting. For example, a pooled screen only provides an association of the perturbation with the phenotype of interest, which may not be an appropriate readout of a complex system [132].

Several groups in the past year have made modifications to the dCas9/gRNA system, circumventing this shortcoming of pooled screens [128,130,131]. Each group also made use of single cell sequencing (scRNA-seq) in order to improve the robustness of the screen and downstream analysis. These scRNA-seq-based approaches allow the perturbation and the phenotype of each cell to be measured simultaneously. In particular, the full transcriptome sequencing analysis provides a ‘one-size-fits-all’ assay that can cover a wide range of phenotypes [133]. By removing the need for specific biomarkers, it may become more broadly applicable for investigating viral infections.

Overall, these large scale perturbation approaches are unbiased and especially suitable for identifying associations between diseases and genes with no known function such as many lncRNAs. The CRISPR-dCas9 system is scalable and provides sufficient throughput for handling the large number of lncRNAs that require functional interrogation. While these newer methodologies like gain-of-function, multiplexed library screens, and scRNA-seq have yet to be implemented in the interrogation of lncRNA functions in the context of viral infection, the utilization of this technology will ultimately hasten the discovery of infection-related lncRNAs.

### 3.5. Considerations for Experimentally Validating Specific lncRNAs

Cross-examining the results generated by different methods described above will likely produce a shorter list of lncRNAs of higher interest. The next challenge is to experimentally validate their associated phenotype, e.g., pathway modulation or resistance to viral infection. Validation is especially important when considering the results of in silico analyses; however, confirmation studies should also be performed with regard to large-scale library screening due to the size and complexity of those assays. Though many existing techniques for studying RNA are easily transferable for investigating lncRNAs, there are some specific considerations given the uniqueness of lncRNAs.

One consideration is the subcellular localization of specific lncRNAs. A nuclear lncRNA may function through interactions with DNA methyltransferases, histone modifying enzymes, or other epigenetic machinery [20,31,32,33,34]. Cytoplasmic localization, on the other hand, is often associated with miRNA sponges or protein trafficking [134,135]. NEAT1 and MALAT1 are particularly well-studied examples, which are amenable to fluorescent localization studies [22,136,137]. Fluorescent in situ hybridization (FISH) techniques have been used extensively to show their localization to nuclear paraspeckles. These techniques can be utilized broadly for the study of lncRNA localization, so long as the appropriate sequence information is available [5].

This subcellular localization information will also inform the choice of experimental techniques. As described, inhibition studies are useful for studying candidate lncRNAs. RNAi, in the form of siRNA, degrades target RNA via DICER and has been used in many instances to inhibit the expression of lncRNAs [112]. Another type of RNAi, antisense oligonucleotides (ASOs), binds target RNA and promotes degradation via RNase H. Unlike other RNAi, these small molecules pass freely through the cell membrane, i.e., without the assistance of transfection reagents, and are capable of entering the nucleus. The ability to pass through the nuclear envelope makes them ideal for targeting lncRNAs, the majority of which localize in the nucleus. siRNA has been reported to be less effective for the knockdown of nuclear targets even though DICER and the RNAi machinery have been shown to be active in the nucleus [138]. A comprehensive analysis found that both siRNAs and ASOs are capable of inhibiting the expression of lncRNAs [113]. As expected, ASOs were more effective against nuclear lncRNA, while siRNAs were more effective against cytoplasmic lncRNAs, and each was effective against lncRNA that localized to both the cytoplasm and the nucleus [113]. Ultimately, the authors concluded that a combination of siRNA and ASOs may be the best method to ensure that the target is sufficiently inhibited [113]. Alternatively, CRISPRi (see Section 3.4) or the traditional CRISPR-Cas9 are effective methods for the inhibition of lncRNA expression [122,139].

The complexity of genomic regions of interest and the ability to target specific lncRNAs in a given region are also important considerations for validation studies. CRISPR inhibition or activation will potentially influence the expression of overlapping or neighboring coding genes, making it difficult to single-out the effects of a specific lncRNA. Goyal et al. [138] illustrate this point well by demonstrating that the lncRNA LOC389641, which arises from a bidirectional promoter of TNFRSF10A, can be specifically inhibited by RNAi. CRISPRi, in this case, down regulates the expression of both genes [138]. Alternatively, the effect of a non-coding RNA may be independent of the transcript, but rely on the act of transcription to modulate a target [114]. In such instances, RNAi would have no functional effect even though it may inhibit the expression of the transcript. To that end, CRISPR-Cas9 would be a more effective system. However, targeting lncRNA with incomplete genomic annotations with the CRISPR-Cas9 system may prove difficult, if not impossible [138]. This outlook will improve as more detailed annotations of non-coding RNA regions are gathered. On the other hand, it is likely that in many instances the specificity of CRISPR systems will not be sufficient and the development of novel methods will be required to adequately modulate lncRNA expression.

Since lncRNA tends to have a lower expression abundance, overexpression might be better suited for lowly expressed lncRNAs and to gain information complimentary to inhibition studies. CRISPRa or ectopic plasmid expression [126,140] are effective methods towards this end. The genomic layout of lncRNAs, such as overlap with coding genes, will prevent ectopic plasmid expression for certain genes. In addition, poorly annotated genes will be difficult to express if the transcription start site (TSS) or the bounds of the gene are unknown. Additionally, overexpression in this manner will not be effective if transcription is the inhibitory event, rather than some function of the lncRNA transcript.

The low expression of lncRNAs is in part due to their cell-type specific expression. Low expression levels can be particularly pronounced when an lncRNA is identified from a heterogeneous cell population, such as blood or tissues. In these cases, it is important to determine the cell types of origin for these lncRNAs. Elucidation of cell type may be done by combining cell staining and FISH techniques coupled with flow cytometry or microscopy. Knowledge of the cell type may also assist in hypothesizing the lncRNA function [141] and designing validation studies.

## 4. Conclusions and Future Direction

Our understanding of the biological relevance of lncRNAs has greatly increased over the past decade. Unfortunately, many of these genes still lack sufficient functional annotation, and their functional role in host-pathogen interactions remains largely unknown. In order to efficiently elucidate the role of these lncRNAs during viral infection, multiple high-throughput methodologies coupled with computational strategies are being utilized to process large quantities of poorly characterized lncRNAs.

RNA-seq has emerged as the most widely used technology for this purpose, which usually results in a large number of lncRNAs significantly associated with viral infection. In order to narrow down these lists of lncRNAs, complementary computational strategies like co-expression network analysis and evolutionary analysis may be leveraged to aid the annotation and prioritization of lncRNA genes. Alternatively, large-scale perturbation screens fueled by rapidly advancing CRISPR-Cas9 techniques are providing novel tools for investigating lncRNA functions in specific areas including viral infection and quickly expanding our knowledge of lncRNA functions. These additional layers of information will reduce the long list of potential interesting lncRNAs to a short list of high-confidence ‘hits’. Ultimately, verifying the mechanism and function of candidate lncRNAs identified by high-throughput strategies requires orthogonal experimental confirmations.

**Figure 1:**
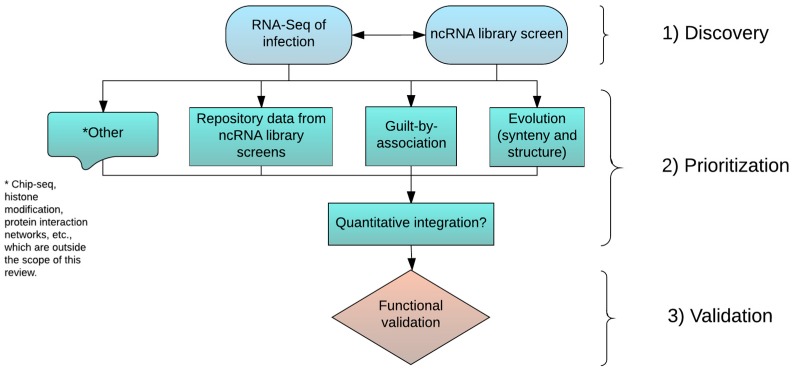
A proposed workflow of three major phases for identifying host lncRNAs that are involved in viral infections. Considering the lack of functional information for lncRNAs in general, the first step will be to survey lncRNAs associated with infection of interest (Discovery phase). Unbiased genome scale approaches like a transcriptome deep sequencing (RNA-seq) analysis of infected samples collected in vitro or in vivo is widely employed. Large-scale lncRNA screening is also emerging as a powerful alternative, but is less applicable due to technical constraints. Once a set of infection-related lncRNAs are identified, the next step is to narrow down the list to a small set of high interest lncRNAs (Prioritization phase), an extremely challenging task. There are multiple computational strategies for the annotation and prioritization of identified lncRNAs based on orthogonal information as indicated. Though not covered here, other types of information, like regulatory elements uncovered by Chip-Seq experiments, histone modification marks, and curated molecular interaction networks can all facilitate the prioritization. However, analytical methods for the quantitative integration of information from different sources need to be developed. This advancement may require community-based collaborative efforts. The last step is to experimentally validate specific candidate lncRNAs (Validation phase), while accounting for the unique characteristics of lncRNAs.

Figure 1 shows a proposed workflow for the identification of key lncRNAs that are most relevant to specific viral infections. The proposed workflow includes three major phases: (1) broad discovery of infection associated lncRNAs; (2) annotation and prioritization of identified lncRNAs; and (3) experimental validation of specific candidate lncRNAs. The most challenging task is to devise efficient learning strategies for ranking high quality lncRNA “hits” by quantitatively combining multiple sources of information. However, to achieve this goal, it is necessary to establish high quality training datasets in which the functions of a sizable number of lncRNAs have been experimentally verified. Simultaneously, compatible high-throughput datasets are needed from the same experimental systems in order to extract predictive features for inferring lncRNA functions. We anticipate that as large scale perturbation techniques mature, some of these lncRNA screen studies will emerge as initial training datasets. Obviously, given the complexities of different high-throughput technologies, developing benchmark training datasets for this purpose requires community-based collaborative efforts.

In summary, the large number of less studied lncRNAs represent a great opportunity for uncovering novel insights into virus-host interactions and potentially new targets for intervention. To fully realize this potential, different high-throughput technologies can be leveraged. While efforts are underway to enable searching putative lncRNAs across large multimodal datasets [142], there is a pressing need to develop specialized computational strategies for prioritizing candidate lncRNAs for downstream experimental validations.

## Figures and Tables

**Table 1:** LncRNAs involved in viral infection.

lncRNA	Encoding Organism	General Function	Specific Function	Infection Type	ID Method	Citation
NEAT1	Host	Scaffold	Nuclear localization, paraspeckle formation	HIV-1, HTNV	1 of 83 lncRNAs profiled in HIV-1-infected Jurkat and MT4 cells	[2,4,24]
NRON	Host	Scaffold	Latency via inhibition of NFAT nuclear translocation	HIV-1	1 of 90 lncRNAs profiled in two human T cell lines	[25,26]
Tmevpg1 (NeST, IfngAS1)	Host	Epigenetics	IFN-gamma-mediated regulation of adaptive immunity	Theiler’s murine encephalo myelitis (TMEV)	Candidate gene from Tmevp3 locus	[27] and reviews by [6,23]
NRAV	Host	Epigenetics	Modulates transcription of ISGs, promotes IAV replication	Influenza A Virus (IAV)	1 of 907 differentially expressed lncRNAs from microarray analysis	[28] and reviews by [6,8]
HIV-expressed antisense lncRNA (ASP-L)	Pathogen	Epigenetics	Epigenetic transcriptional regulation	HIV-1	qPCR	[29]
PAN RNA	Pathogen	Epigenetics	Required for KSHV gene expression, repression of IFN-alpha, IFN-gamma, ISGs	Kaposi’s Sarcoma-associated Herpes-virus (KSHV)	Northern Blot	[30] and reviews by [7,8]
